# Exercise-induced CITED4 expression is necessary for regional remodeling of cardiac microstructural tissue helicity

**DOI:** 10.1038/s42003-022-03635-y

**Published:** 2022-07-04

**Authors:** Robert A. Eder, Maaike van den Boomen, Salva R. Yurista, Yaiel G. Rodriguez-Aviles, Mohammad Rashedul Islam, Yin-Ching Iris Chen, Lena Trager, Jaume Coll-Font, Leo Cheng, Haobo Li, Anthony Rosenzweig, Christiane D. Wrann, Christopher T. Nguyen

**Affiliations:** 1 Cardiovascular Research Center, Massachusetts General Hospital, Charlestown, MA 02129 USA; 2 Martinos Center for Biomedical Imaging, Massachusetts General Hospital, Charlestown, MA 02129 USA; 3 Department of Radiology, University Medical Center Groningen, University of Groningen, Hanzeplein 1, 9713 GZ Groningen, The Netherlands; 4 Harvard Medical School, Boston, MA 02129 USA; 5 Ponce Health Sciences University, School of Medicine, Ponce, PR 00716 USA; 6 Massachusetts General Hospital, Cardiology Division and Corrigan Minehan Heart Center, Boston, MA 02114 USA; 7 McCance Center for Brain Health, Massachusetts General Hospital, Boston, MA 02114 USA; 8 Division of Health Sciences and Technology, Harvard-Massachusetts Institute of Technology, Cambridge, MA 02139 USA; 9 Cardiovascular Innovation Research Center, Heart, Vascular, and Thoracic Institute, Cleveland Clinic, Cleveland, Ohio, 44195 USA

**Keywords:** Cardiac hypertrophy, RNA

## Abstract

Both exercise-induced molecular mechanisms and physiological cardiac remodeling have been previously studied on a whole heart level. However, the regional microstructural tissue effects of these molecular mechanisms in the heart have yet to be spatially linked and further elucidated. We show in exercised mice that the expression of CITED4, a transcriptional co-regulator necessary for cardioprotection, is regionally heterogenous in the heart with preferential significant increases in the lateral wall compared with sedentary mice. Concordantly in this same region, the heart’s local microstructural tissue helicity is also selectively increased in exercised mice. Quantification of CITED4 expression and microstructural tissue helicity reveals a significant correlation across both sedentary and exercise mouse cohorts. Furthermore, genetic deletion of CITED4 in the heart prohibits regional exercise-induced microstructural helicity remodeling. Taken together, CITED4 expression is necessary for exercise-induced regional remodeling of the heart’s microstructural helicity revealing how a key molecular regulator of cardiac remodeling manifests into downstream local tissue-level changes.

## Abstract (web summary)

Expression of transcription factor CITED4 is necessary for exercise-induced regional remodeling of the heart’s microstructural helicity, revealing how a key molecular regulator of cardiac remodeling mediates local tissue-level changes.

## Introduction

Exercise has expansive benefits for the adult heart by protecting it from cardiovascular disease^1^. These beneficial effects arise from changes in metabolism, skeletal muscles, peripheral vessels, and the heart itself^2^. Specifically in the heart, exercise induces cardiac structural remodeling in the form of hypertrophy^2–5^, which occurs with an increase in the length and width of cardiomyocytes^2,3^. Aside from producing beneficial myocardial hypertrophy, some transcription factors and cell proliferation markers have also been implicated in producing cardioprotective effects^2,4,6^. Specifically, CBP/p300-interacting transactivators with E [glutamic acid]/D [aspartic acid]-rich-carboxyl terminal domain 4 (CITED4) is known to play a key role in the exercised-induced cell hypertrophy pathways^7,8^. The upregulated CITED4 is involved in physiological heart growth as a downstream effector of transcriptional pathways resulting in cell growth and proliferation^2,9^. Triggered by exercise, although not exclusive to exercise^7^, CITED4 has shown to globally increase in the cardiac tissue as measured by quantitative polymerase chain reaction (qPCR)^2,7^. Importantly, studies using conditional deletion of CITED4 in cardiomyocytes have demonstrated that it is necessary for adaptive cardiac remodeling in response to exercise^7^.

This differs from the pathological remodeling in heart failure where the length of cardiomyocytes increases disproportionately to width^10^. Structural remodeling is a complex yet dynamic process triggered by changes in the cardiac workload which cause either physiological or pathological hypertrophic growth resulting in a spatially altered architecture of the myocardium^1^. While previous studies have confirmed that exercise-induced cardiomyogenesis can naturally occur within mature mammalian hearts leading ultimately to improved cardiac function^1^, maladaptive growth has been linked to an increased risk of heart failure^11–14^. This indicates that the therapeutic abilities of exercise are not specific to cell growth alone but may result in potential downstream changes to tertiary tissue structures that form the microstructural helicity of the heart. The heart’s microstructural helicity has been predominantly characterized tediously through complex histological sectioning^15^ revealing cardiomyocytes are helically oriented smoothly transitioning from left-handedness to right-handedness throughout the transmural tissue layers of the myocardium. Owing to the challenges to performing such complex histology, there has yet to be studies that elucidate the potential impact of exercise-induced molecular mechanisms on the heart’s microstructural helicity.

Recent studies have shown that the heart’s microstructural helicity can be non-invasively quantified by diffusion tensor magnetic resonance imaging (DT-MRI) in mice^16–18^ and patients^19–23^. Detecting the spatial distributions of the diffusion of water molecules enables DT-MRI to map the underlying orientation of cardiomyocytes in each imaging voxel^24,25^. Furthermore, DT-MRI can uncover these microstructural changes to aid in the detection of myocardial fibrosis^21,26–29^ and has also been used to assess myocardial regeneration and microstructural changes by cell-based therapies^30,31^. In addition, previous studies have shown strong utility of DT-MRI in detecting cardiac structural changes associated with hypertrophic cardiomyopathy (HCM) and ventricular arrythmias^22^ demonstrating the significant clinical and scientific potential of this tool. DT-MRI is well poised to characterize the microstructural impact of exercise and can map how exercise-induced hypertrophy manifests at the microstructural level.

Spatially linking the molecular mechanism of CITED4 to microstructural helical remodeling would require meticulous sectioning of the heart to perform thousands of qPCR experiments. However recently, new approaches to RNA in situ hybridization (RNA-FISH) have enabled the quantitative analysis of the spatial distribution of gene expression^32^. RNA-FISH could be used to map the spatial distribution of CITED4 as a physiological proliferation marker to further elucidate its role in microstructural tissue alterations in exercise-induced cardiac remodeling. Furthermore, counterstaining for nuclei with DAPI would allow quantitative measurements of CITED4 expression for a given region of interest.

In this study, we aim to define the spatial interactions between the exercise-induced molecular mechanism of CITED4 with tissue-level microstructural helical remodeling in exercised and sedentary mice. By imploring two novel imaging technologies, RNA-FISH and DT-MRI, we can efficiently interrogate the whole heart while also spatially co-registering quantifications of CITED4 expression with myocardial microstructural tissue helicity. Furthermore, we used cardiomyocyte-specific CITED4 knock out mice (C4KO) to demonstrate the crucial role CITED4 plays in necessitating these microstructural changes following exercise.

## Results

### Exercise from wheel running

**Fig. 1:**
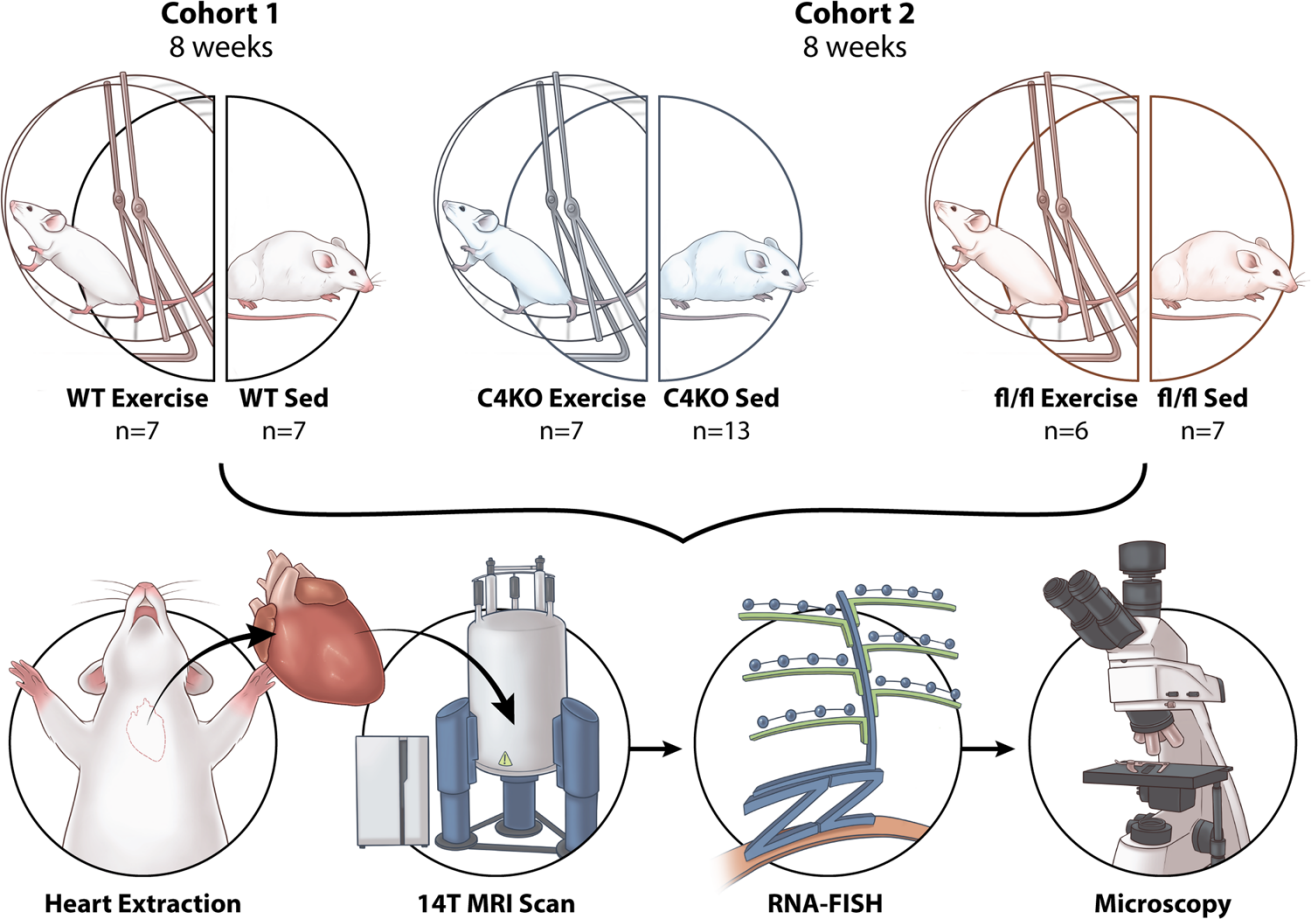
Characterization of the experimental set-up. Cohort 1 consisted of wild-type sedentary (*n* = 7) and wild-type exercise (*n* = 7) mice. Cohort 2 included cardiomyocyte-specific CITED4 knock out (C4KO) exercise (*n* = 7), C4KO sedentary (*n* = 13), fl/fl exercise (*n* = 6) and fl/fl sedentary (*n* = 7) mice. These groups were housed for 8 weeks, and the exercise group had free access to a running wheel. The hearts were perfused and excised after the 8-week holding period. The hearts were imaged using 14 T magnetic resonance imaging (MRI) followed by RNA-FISH of a whole axial slice of the left ventricle. These slides were imaged and analyzed with a fluorescent light microscope.

**Fig. 2:**
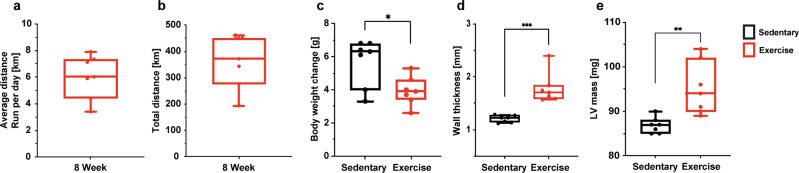
Wheel running promotes significant changes in body weight, heart mass, and thickness. **a** The average distance cohort 1 ran per day in kilometers (km). **b** The average total km cohort 2 ran through 8-weeks. **c** The body weight changes between the sedentary and exercise groups of cohort 1. **d** The difference in wall thickness between the sedentary and exercise groups of cohort 1. **e** The difference in left ventricular (LV) mass between the exercise and sedentary groups. Unpaired two-tailed *t*-test *P < 0.05, **P < 0.01, ***P < 0.001. Data are presented min to max. Within each box, horizontal black and red lines denote median values; boxes extend from the 25th to the 75th percentile of each group’s distribution of values.

Free wheel-running was used to exercise wild-type mice (*n* = 7), C4KO mice (*n* = 7) and fl/fl mice (*n* = 6) to induce cardiac remodeling while sedentary groups of wild-type mice (*n* = 7), C4KO mice (*n* = 13) and fl/fl mice (*n* = 7) were not provided access to running wheels (Fig. 1). The average cumulative total kilometers ran by the exercise group up to 8 weeks was 371.9 [277.0–450.8] km. The average distance run per day by the end of the 8 weeks of the exercise was 6.028 [4.397–7.336] km/day (Fig. 2a, b). Over the eight-week period, the sedentary group had a significantly greater increase in body weight in comparison to the exercise group (*p* = 0.017) (Fig. 2c). Analyses of the wall thickness of the left ventricle and left ventricular (LV) mass in the exercise group demonstrated significant increases (47.7%, *p* = 0.0002 and 9.5%, *p* = 0.004 respectively) recapitulating previously described exercise-induced cardiac remodeling (Fig. 2d, e).

### Exercise induces microstructural remodeling of regional cardiac tissue helicity

**Fig. 3:**
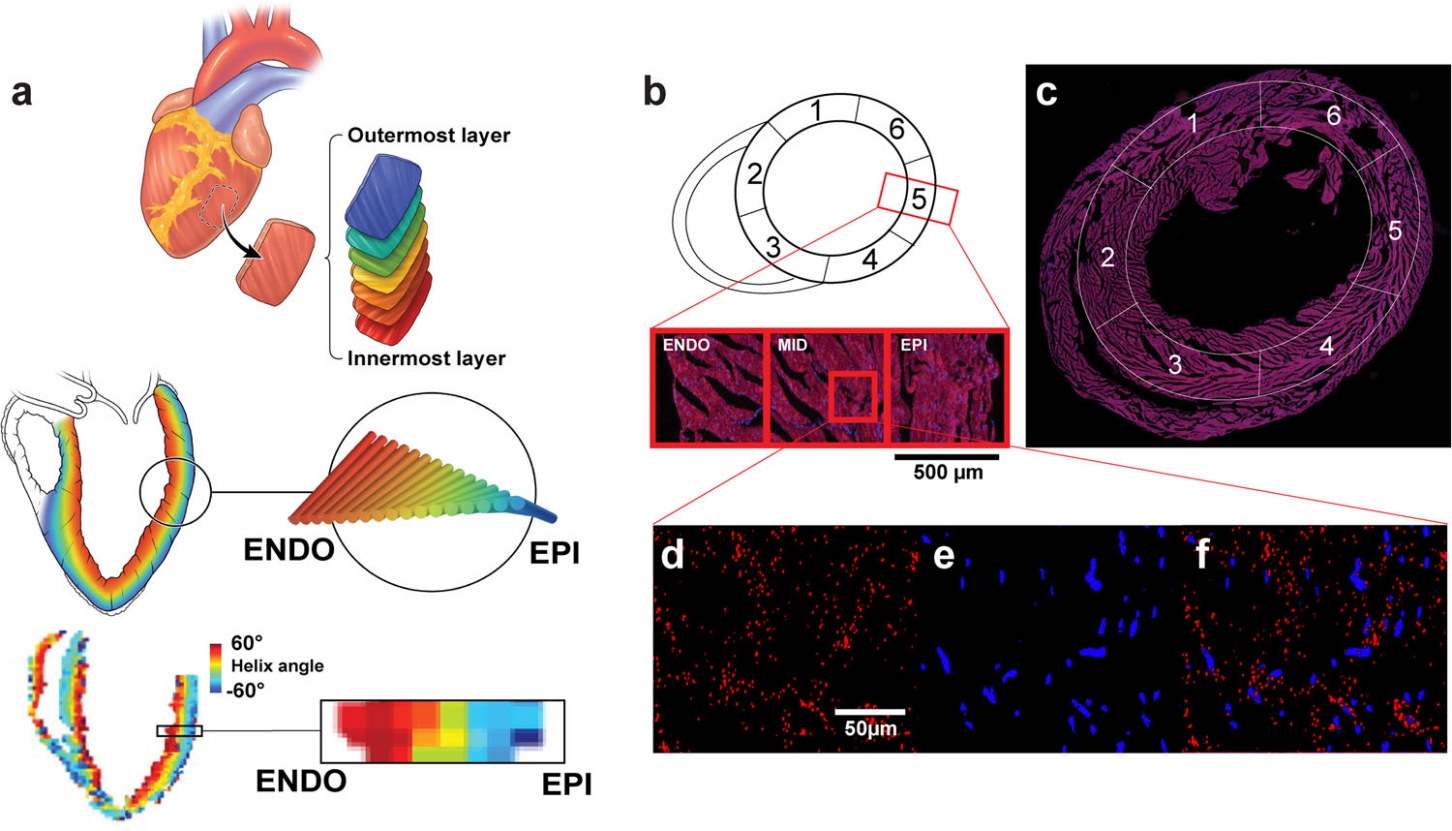
Helix angle analysis and RNA-FISH imaging and quantification of exercise and sedentary mouse hearts. **a** Helix angles change from the endocardium to epicardium between sedentary and exercised mice. **b** A visual representation of the location of the six AHA sections and a zoomed in images of the complete cross-section across each transmural layer. **c** Complete image of sectioned mouse tissue with AHA sections overlayed. **d** CITED4 signal isolated within mid-myocardium. **e** DAPI signal isolated and magnified in the mid-myocardium. **f** CITED4 and DAPI signal imaged from mid-myocardium overlayed.

**Fig. 4:**
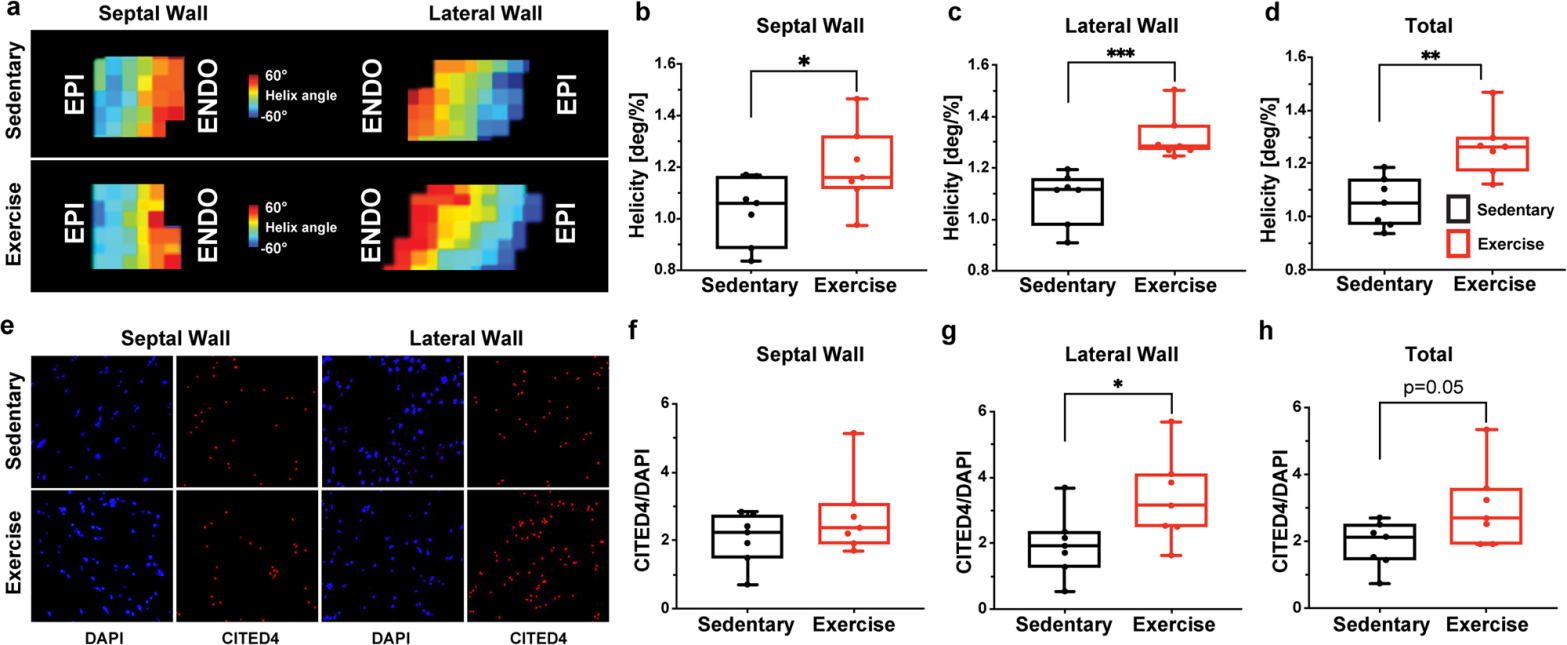
Wheel running in mice promotes physiological remodeling in the heart in coordination with spatial upregulations of CITED4. **a** Representative DT-MRI images of helicity changes from the endocardium to epicardium between sedentary and exercised mice in cohort 1. **b**–**d** Helicity differences within the septum, lateral wall and averaged across all AHA sections. **e** Magnified representative images of DAPI and CITED4 within the septal and lateral wall regions within exercised and sedentary mice. **f**–**h** CITED4 signal in sedentary and exercised mice within the septum, lateral wall and averaged across all AHA sections. Unpaired two-tailed *t*-test. *P < 0.05, **P < 0.01, ***P < 0.001. Data are presented min to max. Within each box, horizontal black and red lines denote median values; boxes extend from the 25th to the 75th percentile of each group’s distribution of values.

After 8 weeks, the sedentary and exercise mouse hearts were perfused and extracted for non-invasive ex vivo DT-MRI to reveal the heart’s tissue microstructure. Fiber orientation was mapped for each voxel, and regional cardiac tissue helicity was calculated across the transmural wall for each American Heart Association (AHA) segment as the gradient of the helix angle^15^ normalized to the percent heart wall depth from the endocardium (innermost layer) to the epicardium (outermost layer) (Fig. 3a). Total cardiac tissue helicity was significantly (*p* = 0.0024) increased (19.9%) in the exercise cohort compared with the sedentary cohort (Fig. 4d). Regional cardiac tissue helicity analyses revealed significant (*p* = 0.048) increases (17.1%) within the septal region (AHA segment 2 and 3) and even greater significant (*p* = 0.0007) increases (21.5%) in the lateral wall (AHA segments 5 and 6) when comparing exercise with sedentary cohorts (Fig. 4a–d). This underlying remodeling of cardiac tissue helicity in the exercise cohort was predominantly driven by the increase in the helix angle of the endocardial layer (Fig. 4a).

### Cardiac regional heterogeneity in exercise-induced modulation of CITED4 expression

Following DT-MRI, the intact hearts were sectioned and then stained during RNA-FISH for CITED4 and DAPI. These sections were imaged under fluorescent light microscopy and the tissue level CITED4 expression normalized to DAPI nuclei counts was quantified (Fig. 3b–f). The exercise group revealed a trend towards an increased (59%) total CITED4*/*DAPI ratio (Fig. 4h, *p* = 0.051). A regional analysis revealed an increased (71.7%) CITED4/DAPI ratio in the lateral wall (including AHA segments 5 and 6) between the exercise and sedentary groups (Fig. 4e, g, *p* = 0.048). However, while there was also an increase in the CITED4/DAPI ratio (32.9%) of the septal region (including AHA segments 2 and 3), the difference between the exercise and sedentary group was insignificant (Fig. 4e, f, *p* = 0.22). Further spatial comparisons of the CITED4/DAPI ratio within the epi-, mid- and endocardium of each AHA section revealed further heterogeneity of CITED4 expression (Supplemental Figs. 1a–c, 2). Significant increases in the CITED4/DAPI ratio were present within varying AHA sections within the three transmural layers (Supplemental Figs. 1a–d, 2).

### Exercise-induced modulation of CITED4 expression is spatially linked to microstructural remodeling of cardiac tissue helicity

**Fig. 5:**
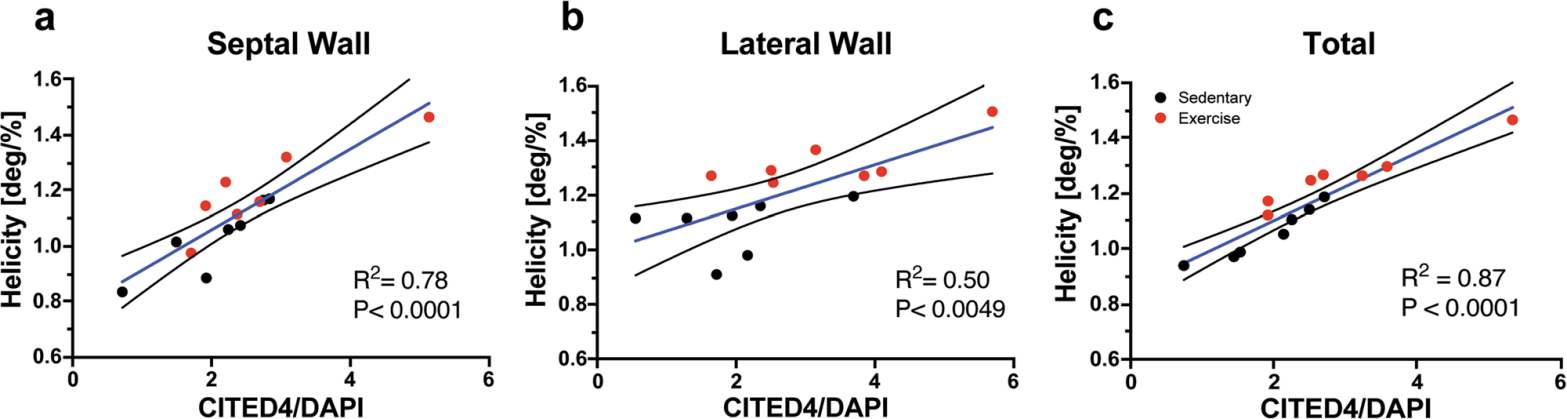
Regional helicity changes are significantly correlated with CITED4/DAPI expression. **a**–**c** Simple linear regressions of CITED4/DAPI signal and helicity within the septum, lateral wall and averaged across all AHA sections. Black dots represent sedentary animals and red dots represent exercised animals. The blue line represents the total simple linear regression of both groups. *P < 0.05, **P < 0.01, ***P < 0.001. Data presented with mean and 95% confidence interval.

To investigate the spatial relationship between CITED4 expression and microstructural remodeling of cardiac tissue helicity, interclass correlations were performed on septal/lateral wall regional segments and total tissue level. Moderate correlations were found within the septal and lateral regions between CITED4/DAPI ratio and cardiac tissue helicity (*R*^2^ = 0.78, *p* < 0.0001 and *R*^2^ = 0.50, *p* < 0.0001 respectively) (Fig. 5). Substantial correlations were found between the total CITED4/DAPI ratio and total cardiac tissue helicity (*R*^2^ = 0.87, *p* < 0.0001).

### Exercised C4KO mice exhibit blunted microstructural remodeling of cardiac tissue helicity

**Fig. 6:**
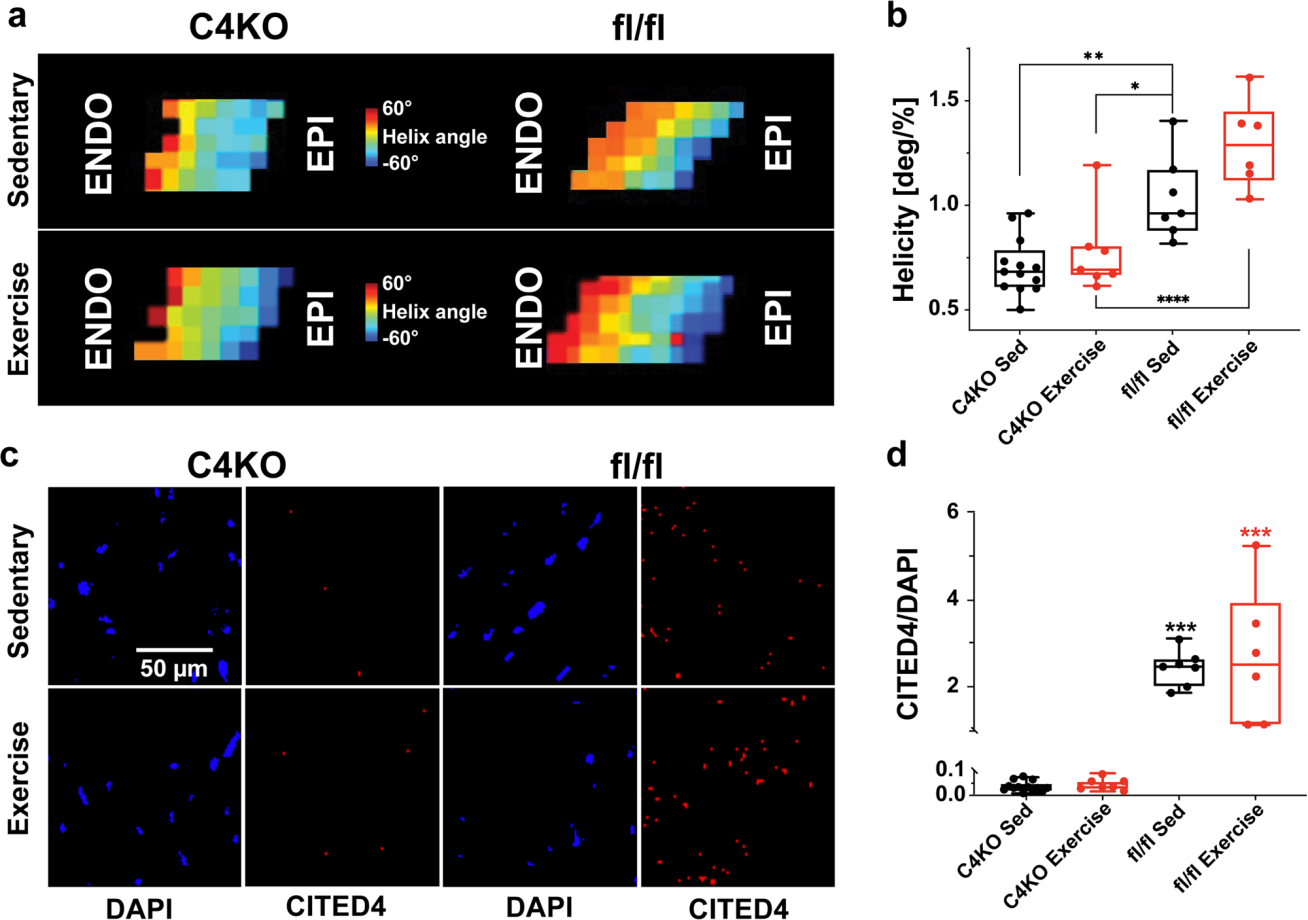
Cardiomyocyte-specific knock out of CITED4 impacts regional tissue helicity changes. **a** Helix angle changes differ significantly between groups. **b** An analysis of helicity between all groups of cohort 2. **c** Images of DAPI and CITED4 staining of representative mice from cohort 2. **d** Quantification of CITED4/DAPI using RNA-FISH with cohort 2. Two-way ANOVA used for multivariant analyses *P < 0.05, **P < 0.01, ***P < 0.001. Data are presented min to max. Within each box, horizontal black and red lines denote median values; boxes extend from the 25th to the 75th percentile of each group’s distribution of values.

To understand whether CITED4 was necessary for microstructural remodeling, we performed DT-MRI and RNA-FISH on mice with cardiomyocyte-specific deletion of CITED4 (C4KO). Cardiac tissue helicity was significantly reduced in both C4KO sedentary and exercise groups compared to the fl/fl sedentary group (−31.7%, *p* = 0.0024) and fl/fl exercise group (−40.3%, *p* < 0.0001, Fig. 6a, b). In the fl/fl group, exercise produced a trending increase in cardiac tissue helicity similar to the wild types in previous experiments (25.1%, *p* = 0.06). We measured the levels of CITED4 expression in these groups to ensure that the C4KO and fl/fl groups were expressing CITED4 as expected. The cardiomyocyte-specific knock out of CITED4 produced a negligible expression of CITED4 (Fig. 6c, d). Meanwhile, the expression of CITED4 in the fl/fl was comparable to cohort 1 (Fig. 6c, d).

## Discussion

In this study, the spatial distribution and upregulation of the physiological growth marker CITED4 were determined in exercised cardiac mouse tissue using RNA-FISH and compared with DT-MRI-based microstructural tissue biomarkers. The trend towards a total increase in the CITED4/DAPI ratio in the exercised heart tissue was in line with previous reports using qPCR^2,7,8^. However, RNA-FISH also allowed for a more targeted spatial analysis which revealed increased CITED4/DAPI ratios primarily within the lateral wall. Furthermore, the ex vivo anatomical cardiac MRI of both the sedentary and exercised hearts confirmed the increase in left ventricular mass by exercise^1,2^ and the myocardial microstructural characterization revealed the microstructural alterations characterized by helicity within the left ventricle of the exercised mice. The direct spatial correlation of these microstructural tissues and molecular changes could provide further insight into how molecular expression can manifest into gross anatomical and physiological remodeling. To the best our knowledge, this is the first study that investigates the interplay between regional gene expression and microstructural tissue remodeling yielding insight on how tissue level remodeling may manifest from molecular mechanisms.

DT-MRI is a powerful tool to determine small scale fiber orientation changes associated with the disease pathology^30,31^. In this study, DT-MRI of the exercised mice revealed similar microstructural changes to previous results in myocardial infarction mouse models treated with cell therapy injections^31^, which included significant changes to the helicity of the myocardial fibers. However, in this study, these changes to microstructural helicity are tied directly to exercise-induced structural remodeling, which is shown to result in improved outcomes following cardiac injury and disease^30^. These exercise-induced changes to helicity shown in this study further demonstrate the structural cardiac remodeling benefits of exercise, and in conjunction with past work^30^, help to demonstrate the structural changes produced by exercise that may be cardioprotective. Given DT-MRI can be performed in vivo and non-invasively^19–23^, potentially these CITED4-induced myocardial microstructural changes could be explored to see if they are cardioprotective in a rescue preclinical study.

Conventional qPCR has routinely been used as the standard procedure for determining total CITED4 expression^1,2,8^. However, in this study, we were able to demonstrate the difference in the spatial distribution of CITED4 expression across the six different AHA regions of the heart and within the three different transmural layers using RNA-FISH. Specifically, increases in CITED4 within the lateral wall correlate with cardiac remodeling revealed by DT-MRI-based microstructural changes. Although, qPCR already showed significant increases in CITED4 expression within exercised cardiac tissue^2,7^, the use of RNA-FISH-based spatial information in this study helped to directly link the microstructural tissue modifications with the local transcriptional changes. The implications of this CITED4 local expression may serve to guide more targeted delivery of CITED4^7^ or other modifiers to cardiac tissue, such as Irisin^33,34^.

While CITED4 is known to play a role in tissue proliferation and hypertrophy^2,7–9^, it is inversely regulated by the transcription factor C/EBPβ^2^ and manipulation of CITED4 levels has been shown to impact cardiomyocyte proliferation^8^. Following exercise, a reduction in C/EBPβ leads to an increase in cell growth and division^2^ and at the same time C/EBPβ may also be involved in the induction of the PGC1α pathway, which prevents cardiac dysfunction following hypertrophy^2,8^. The inhibition of CITED4 by C/EBPβ led to the hypothesis that C/EBPβ’s impact on reducing physiological growth may be directly linked to the inhibition of CITED4^8^. Data presented within this study, as well as recent studies, seem to further confirm this hypothesis^2,7^. Furthermore, this CITED4 pathway provides a potential link to the microstructural changes revealed through DT-MRI. The specific changes in helicity present between the exercise and sedentary groups might be explained, in part, by these exercised-induced molecular changes.

The specific role of CITED4 in regulating gene transcription, appears to impact exercise-induced cardiac growth, as it has been found to be a physiological marker for cardiac growth which is also seen in the increase in cardiac wall thickness and LV mass in this study^2,8^. Although more studies must be performed to further elucidate this pathway, it appears C/EBPβ and CITED4 are intrinsically tied to the production of exercise-induced cardiac growth^2,8,35^. Exercise specifically upregulates CITED4, while downregulating C/EBPβ to produce beneficial cardiomyocyte growth and tissue remodeling from micro to macro and from structural to functional^2,8,35^. In this study, DT-MRI analyses confirm that the trends in increased CITED4 expression occur in conjunction with the widespread structural changes marked by increased helicity. Our results specifically indicate an increased amount of CITED4 expression within the lateral wall of the left ventricle. This upregulation of CITED4 within the lateral wall of the left ventricle could also help to elucidate the mechanism for the specific lateral wall increases in tissue volume found with DT-MRI.

Utilizing the CITED4 KO mice, we demonstrated the necessary role that CITED4 plays in cardiac microstructural changes the following exercise thereby addressing a key gap in knowledge between the molecular mechanism of exercise and how it manifests into tissue level changes. In mice with cardiac-specific deletion of CITED4, there were significant differences in the DT-MRI biomarker of helicity, indicating that CITED4 is required for cardiac structural changes to take place. In addition, without active expression of CITED4, exercise resulted in no differences within these DT-MRI markers, further proving that CITED4 expression is necessary for exercise-induced structural changes. Meanwhile, we demonstrated that active expression of CITED4 coupled with exercise, results in significant structural remodeling of the cardiac tissue in both the wild-type mice of cohort 1 and the fl/fl mice of cohort 2. These results highlight the necessary and required role CITED4 plays in cardiac remodeling following exercise. Our results indicate that CITED4 also plays an essential role in the baseline helicity formation as shown by the significant reduction in helicity in both the C4KO sedentary and exercise groups compared to the fl/fl sedentary. Presumably, helicity should have been returned to the same level as the fl/fl sedentary but was detrimentally reduced when cardiomyocyte-specific CITED4 was knocked out.

Finally, we want to emphasize that the novel imaging technologies used in the study are generalizable and can be used to spatially link other molecular mechanisms to microstructural tissue remodeling. The novel imaging technologies could also be used to investigate other cardiovascular diseases as well as the impact of targeted molecular therapies such as mRNA. We believe our study marks a potential new platform to examine how molecular mechanisms manifest into downstream tertiary tissue structures, which may play a critical role in how to scale up the impact of molecular therapeutics.

However, this study has several potential limitations. The small sample size of the exercise and sedentary mouse groups is a possible limitation. Despite this limitation, a significant correlation was found between the upregulation CITED4 and changes in myocardial microstructure. Comparing cohort 1 and 2 had limitations, as in cohort 2 we only had access to mid-ventricular slices of the cardiac tissue. This prevented us from doing some of the same analyses we did for the first cohort of animals. Further, the knockdown of CITED4 in cohort 2 was specific only to the cardiomyocytes. However, this should not have impacted the results as CITED4 is expressed at very low levels in fibroblasts and other noncardiomyocytes^7,8^. Finally, the co-registration of CITED4 expression analyzed through RNAscope and helicity measured with DT-MRI could be a possible limitation. Future studies should pursue a more advanced method of co-registration between mRNA expression and DT-MRI markers.

Overall, this study confirmed the hypothesis that exercise leads to left ventricular microstructural changes in the heart’s helicity and that CITED4 plays a necessary role in this process. We demonstrated that the expression of CITED4 following exercise differs by cardiac regions, which can explain the adaptive patterns of cardiac remodeling determined by cardiac DT-MRI. Furthermore, we showed deletion of the CITED4 expression in transgenic mice leads to complete prohibition of the exercise-induced remodeling of the heart’s microstructure. These findings serve as a fundamental basis for understanding exercise-induced hypertrophic changes at a molecular and microstructural level and motivate further evaluation of exercise and cardiovascular health. Future studies should explore the impacts of exercise on CITED4 using large animal models to gain a better understanding of how exercise may influence cardiac remodeling in humans.

## Methods

### Animal studies

All animal procedures were approved by the Institutional Animal Care and Use Committee (IACUC) of our institute. Mice were maintained in a specific pathogen-free environment at an in-house animal facility. The mice were kept under 12-h light/12-h dark cycles at a constant room temperature (22 °C). Mice had access to a standard diet of food and water ad libitum.

### Study design

Two cohorts of mice were used in this study. In the first cohort, A total of fourteen seven-week-old male wild-type C57BL/6J mice (Jackson Laboratory, USA) were housed in the same facility. The mice were housed individually in cages with (exercise, *n* = 7) or without (sedentary, *n* = 7) free access to running wheel (Starr Life Sciences, USA) in the same facility (Fig. 1). The running activity of these seven mice was tracked using a revolution counter that tracked running activity data every hour. The mice were weighed again after four and eight weeks.

In the second cohort, CITED4 knockout (KO) mice were generated by breeding CITED4 floxed mice (fl/fl) and hemizygous αMHC-Cre mice as described previously^7^. Only male CITED4 KO mice and its control littermates (fl/fl mice) were used and randomized into four groups: CITED4 KO sedentary (*n* = 13), CITED4 KO exercise (*n* = 7), CITED4 fl/fl sedentary (*n* = 7), and CITED4 fl/fl exercise (*n* = 6). The mice from these groups were 8–12 weeks old. All mice were held in the same room in the animal facility. The running group (*n* = 13) was housed in cages with stainless steel running wheels (Starr Life Sciences, USA) (Fig. 1). The remaining mice (*n* = 20) belonged to the sedentary group and were housed identically, except without access to a running wheel.

After eight weeks of running, the mice were euthanized using isoflurane anesthesia followed by cardiac puncture and perfused with 4% paraformaldehyde (PFA, Electron Microscopy Sciences, USA). Hearts were extracted and stored in 4% PFA. For cohort 1, MRI scans were taken of the whole heart. For cohort 2, a mid-ventricular slice of 1 mm width was taken for imaging and sectioning.

### MRI acquisition

After initial perfusion, the hearts and sections were placed in a 15-ml Falcon tube and submerged in perfluoropolyether (Solvay Specialty Polymers, Italy) for 1 month +/− 3 weeks. Diffusion tensor magnetic resonance imaging (DT-MRI) of the mouse hearts was performed on a 14 T MRI scanner (Bruker Avance III HD, PV 6.0.1) with a 20 mm birdcage coil. Twelve diffusion-weighted (*b* = 500 s/mm^2^) and four non-diffusion-weighted (*b* = 0 s/mm^2^) single spin-echo MRI images were acquired with the following imaging parameters: repetition time  = 1500 ms, echo time = 12.37 ms, number of averages = 3, acquisition matrix = 128 × 128 × 80, spatial resolution = 140 μm × 140 μm, slice thickness = 140 μm, receiver bandwidth = 7100 Hz, diffusion duration = 7 ms, total scan time 6 h 24 m 0 s. DT-MRI was acquired in a stack of short-axis reaching from the base to the apex and the 4- and 2-chamber long axis.

### MRI image analysis

The DT-MRI-derived cardiac helicity serves as the readout parameters of myofiber organization and architecture investigated at the three transmural zones (epicardium, mid-myocardium and endocardium). ﻿ Pixel-wise values of helix angle were calculated using custom software in Matlab (Mathworks, Natick, MA)^36^. Tensor reconstruction was calculated using a modified weighted least squares fit. Mean LV helicity was calculated for each short-axis slice by automatically segmenting the LV into five transmural concentric rings, following which the slope was extracted from the linear regression of the mean helix angle for each ring against the transmural depth from the endocardium to the epicardium.

### RNA in situ hybridization

Following DT-MRI, the hearts were cleaned with saline and dehydrated within 10, 20, and 30% sucrose solutions. The hearts were then embedded in Optimal Cutting Temperature (OCT) gel (Sakura Finetek INC., USA) and flash frozen before being stored at −80 °C for 24 h. They have subsequently sectioned in 10 μm short-axis slices at the midventricular level on a Leica CM3050 S (Leica Biosystems, Germany) research cryostat and placed on Superfrost microscope slides (Fisherbrand®, (ThermoFisher Scientific, USA). RNAscope® in situ hybridization (Advanced Cell Diagnostics INC., U.S.) was performed on 1 mid-ventricular slice per heart following the manufacturer’s instructions. In short, samples were pretreated by heating the slides at 60 ^o^C for 30 min and post fixed in 4% PFA at 4 ^o^C for 15 min. This was followed by dehydration in 50, 70, and 100% ethyl alcohol (EtOH) for 5 min each and repeated once more with 100% EtOH. After this sample preparation, they were processed with the RNAscope® multiplex fluorescent reagent kit V2 (Advanced Cell Diagnostics INC., U.S.) using the Mm-CITED4 probe (Advanced Cell Diagnostics INC., U.S.). All slides were DAPI (Advanced Cell Diagnostic INC., U.S.) stained prior to cover slipping. Samples were imaged on a Zeiss Axio Imager.A2 microscope at 20× (Carl Zeiss AG, Germany). For cohort 1, images were taken in six separate segments following the American Heart Association (AHA) heart segmentation of a mid-ventricular slice. These six-segment images were obtained by stitching 20× cross-sectional images together using Zen 2.3 Pro software (Carl Zeiss AG, Germany). In cohort 2, single images at 20× were taken from the mid-ventricular slice.

### RNA-FISH image analysis

Images of the RNA-FISH assays were processed and quantified using an in-house thresholding and counting software written in MATLAB (MATLAB 2019a, MathWorks, USA). In cohort 1, three ROIs were created within each of the six segments of each heart that represent the epi-, myo-, and endocardium. For cohort 2, whole images were analyzed. The images were processed to a threshold that accurately isolated and counted CITED4 and DAPI signals and manually adjusted to insure the inclusion of all. This resulted in the number of CITED4 and DAPI signals per AHA segment per epi-, myo- and endocardial transmural layer. The CITED4 expression was normalized by the DAPI count to determine the CITED4 expression per cell nuclei.

### Statistics and reproducibility

Unpaired *t*-tests were used for single group comparisons of normally distributed data. Two-way ANOVA followed by Tukey post hoc was used for multivariant comparisons of normally distributed data. Simple linear regressions were used to assess the correlation of DT-MRI-based microstructural biomarkers with CITED4/DAPI expression ratio. *p* values < 0.05 were considered statistically significant. Statistical analysis was performed using GraphPad Prism8 software (San Diego, CA).

### Reporting summary

Further information on research design is available in the Nature Research Reporting Summary linked to this article.

## Supplementary information

Supplementary Information

Description of Additional Supplementary Files

Supplementary Data 1

Reporting Summary

## Data Availability

The source data for the graphs in the main figures are provided in Supplementary Data 1. The other data that support the findings of this study are available from the corresponding author, upon reasonable request.
