# Author Correction: Exercise-induced CITED4 expression is necessary for regional remodeling of cardiac microstructural tissue helicity

**DOI:** 10.1038/s42003-022-03671-8

**Published:** 2022-07-13

**Authors:** Robert A. Eder, Maaike van den Boomen, Salva R. Yurista, Yaiel G. Rodriguez-Aviles, Mohammad Rashedul Islam, Yin-Ching Iris Chen, Lena Trager, Jaume Coll-Font, Leo Cheng, Haobo Li, Anthony Rosenzweig, Christiane D. Wrann, Christopher T. Nguyen

**Affiliations:** 1 Cardiovascular Research Center, Massachusetts General Hospital, Charlestown, MA 02129 USA; 2 Martinos Center for Biomedical Imaging, Massachusetts General Hospital, Charlestown, MA 02129 USA; 3 Department of Radiology, University Medical Center Groningen, University of Groningen, Hanzeplein 1, 9713 GZ Groningen, The Netherlands; 4 Harvard Medical School, Boston, MA 02129 USA; 5 Ponce Health Sciences University, School of Medicine, Ponce, PR 00716 USA; 6 Massachusetts General Hospital, Cardiology Division and Corrigan Minehan Heart Center, Boston, MA 02114 USA; 7 McCance Center for Brain Health, Massachusetts General Hospital, Boston, MA 02114 USA; 8 Division of Health Sciences and Technology, Harvard-Massachusetts Institute of Technology, Cambridge, MA 02139 USA; 9 Cardiovascular Innovation Research Center, Heart, Vascular, and Thoracic Institute, Cleveland Clinic, Cleveland, Ohio, 44195 USA

**Keywords:** Cardiac hypertrophy, RNA

Correction to: *Communications Biology* 10.1038/s42003-022-03635-y, published online 04 July 2022.

In this article the grant numbers NS087096 and NS117694 relating to NIH and the funding from ‘Hassenfeld Clinical Scholar Award’ for Christiane Wrann were omitted. The original article has been corrected.

