# Supplementary material for: Exercise-induced CITED4 expression is necessary for regional remodeling of cardiac microstructural tissue helicity
Source: Commun Biol. 2022 Jul 4;5:656. doi: 10.1038/s42003-022-03635-y (PMC9253017; doi:10.1038/s42003-022-03635-y)
Supplement: Supplementary file 1 — Supplementary Information [file 42003_2022_3635_MOESM1_ESM.pdf]

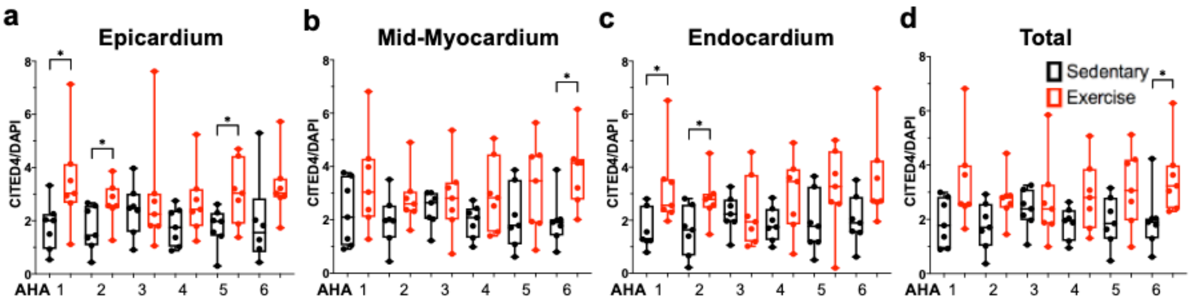

Supplementary Figure 1: CITED4 expression is heterogeneous across different transmural layers and AHA sections (**a-c**) Quantification of CITED4/DAPI using RNA-FISH with mice from cohort 1 across the six AHA sections and three transmural layers; epicardium, mid-myocardium, and endocardium respectively. (**d**) An average of CITED4/DAPI across the three transmural layers within each AHA section. Unpaired two-tailed t-test. *P < 0.05, **P < 0.01, ***P < 0.001. Data are presented min to max. Within each box, horizontal black and red lines denote median values; boxes extend from the 25th to the 75th percentile of each group's distribution of values.

	1		2		3		4		5		6	
EPI	3.03 [2.67-4.14]	2.01 [0.93-2.26]	2.57 [2.48-3.23]	1.45 [1.07-2.56]	2.25 [1.80-3.02]	2.48 [1.57-3.02]	2.43 [1.80-3.20]	1.75 [1.02-2.39]	3.04 [1.90-4.42]	1.98 [1.43-2.30]	3.03 [2.88-3.59]	1.55 [0.81-2.84]
	p=0.049 *		p=0.044 *		p=0.60 ns		p=0.12 ns		p=0.032 *		p=0.13 ns	
MID	3.04 [2.10-4.30]	2.10 [1.04-3.66]	2.60 [2.32-3.07]	1.97 [1.32-2.54]	2.81 [2.01-3.41]	2.64 [2.03-3.02]	2.83 [1.55-4.46]	2.08 [1.32-2.43]	3.46 [1.88-4.42]	1.79 [1.08-3.50]	4.13 [2.76-4.28]	1.91 [1.41-2.02]
	p=0.20 ns		p=0.14 ns		p=0.48 ns		p=0.10 ns		p=0.18 ns		p=0.011 *	
ENDO	2.56 [2.30-3.55]	1.28 [1.22-2.56]	2.79 [2.46-3.00]	1.64 [0.66-2.59]	1.94 [1.19-3.71]	2.32 [1.95-2.79]	3.46 [1.84-3.93]	1.74 [1.25-2.43]	3.27 [2.62-4.61]	1.77 [1.18-3.27]	2.73 [2.68-4.26]	1.90 [1.53-2.88]
	p=0.030 *		p=0.029 *		p=0.91 ns		p=0.086 ns		p=0.12 ns		p=0.055 ns	
Total	2.57 [2.49-3.99]	1.78 [0.92-2.83]	2.80 [2.42-2.88]	1.70 [1.02-2.57]	2.38 [1.83-3.28]	2.39 [1.97-3.10]	2.80 [1.67-3.86]	1.96 [1.20-3.10]	3.06 [1.96-4.21]	1.85 [1.28-2.80]	3.23 [2.41-3.99]	1.90 [1.30-2.03]
	p=0.063 ns		p=0.051 ns		p=0.62 ns		p=0.081 ns		p=0.080 ns		p=0.035 *	

Supplementary Figure 2: CITED4 expression is heterogeneous across different transmural layers and AHA sections. Medians and IQR of CITED4/DAPI of cohort 1 for each transmural layer and AHA section. The exercise group is represented within the left-hand column of each AHA section with the sedentary group in the right-hand column. Unpaired two-tailed t-test *P < 0.05, **P < 0.01, ***P < 0.001.
