# Supplementary material for: Exercise-induced CITED4 expression is necessary for regional remodeling of cardiac microstructural tissue helicity
Source: Commun Biol. 2022 Jul 4;5:656. doi: 10.1038/s42003-022-03635-y (PMC9253017; doi:10.1038/s42003-022-03635-y)
Supplement: Supplementary file 4 — Reporting Summary [file 42003_2022_3635_MOESM4_ESM.pdf]

nature portfolio

Corresponding author(s): Dr. Christopher Nguyen

Last updated by author(s): 6/22/2022

Reporting Summary

Nature Portfolio wishes to improve the reproducibility of the work that we publish. This form provides structure for consistency and transparency in reporting. For further information on Nature Portfolio policies, see our Editorial Policies and the Editorial Policy Checklist.

Statistics

For all statistical analyses, confirm that the following items are present in the figure legend, table legend, main text, or Methods section.

n/a	Confirmed	
☐	☒	The exact sample size (*n*) for each experimental group/condition, given as a discrete number and unit of measurement
☐	☒	A statement on whether measurements were taken from distinct samples or whether the same sample was measured repeatedly
☐	☒	The statistical test(s) used AND whether they are one- or two-sided<br>*Only common tests should be described solely by name; describe more complex techniques in the Methods section.*
☐	☒	A description of all covariates tested
☐	☒	A description of any assumptions or corrections, such as tests of normality and adjustment for multiple comparisons
☐	☒	A full description of the statistical parameters including central tendency (e.g. means) or other basic estimates (e.g. regression coefficient) AND variation (e.g. standard deviation) or associated estimates of uncertainty (e.g. confidence intervals)
☐	☒	For null hypothesis testing, the test statistic (e.g. F, t, r) with confidence intervals, effect sizes, degrees of freedom and P value noted<br>*Give P values as exact values whenever suitable.*
☒	☐	For Bayesian analysis, information on the choice of priors and Markov chain Monte Carlo settings
☒	☐	For hierarchical and complex designs, identification of the appropriate level for tests and full reporting of outcomes
☒	☐	Estimates of effect sizes (e.g. Cohen's *d*, Pearson's *r*), indicating how they were calculated

Our web collection on statistics for biologists contains articles on many of the points above.

Software and code

Policy information about availability of computer code

Data collection	Diffusion tensor magnetic resonance imaging (DT-MRI) of the mouse hearts was performed on a 14T MRI scanner (Bruker Avance III HD, PV 6.0.1, Billerica, MA). RNAscope samples were imaged on a Zeiss Axio Imager.A2 microscope (Carl Zeiss AG, Germany). The images were stitched together using Zen 2.3 Pro software (Carl Zeiss AG, Germany).
Data analysis	The DT-MRI values were calculated using custom software in Matlab (Mathworks, Natick, MA). Images of the RNA-FISH assays were processed and quantified using MATLAB (MATLAB 2019a, MathWorks, USA). Statistical analysis was performed using GraphPad Prism8 software (San Diego, CA).

For manuscripts utilizing custom algorithms or software that are central to the research but not yet described in published literature, software must be made available to editors and reviewers. We strongly encourage code deposition in a community repository (e.g. GitHub). See the Nature Portfolio guidelines for submitting code & software for further information.

Data

Policy information about availability of data

All manuscripts must include a data availability statement. This statement should provide the following information, where applicable:

- Accession codes, unique identifiers, or web links for publicly available datasets
- A description of any restrictions on data availability
- For clinical datasets or third party data, please ensure that the statement adheres to our policy

The source data for the graphs in the main figures are provided in supplementary information. The data that support the findings of this study are available from the corresponding author, upon reasonable request.

Human research participants

Policy information about studies involving human research participants and Sex and Gender in Research.

Reporting on sex and gender	N/A
Population characteristics	N/A
Recruitment	N/A
Ethics oversight	N/A

Note that full information on the approval of the study protocol must also be provided in the manuscript.

Field-specific reporting

Please select the one below that is the best fit for your research. If you are not sure, read the appropriate sections before making your selection.

☒ Life sciences ☐ Behavioural & social sciences ☐ Ecological, evolutionary & environmental sciences

For a reference copy of the document with all sections, see nature.com/documents/nr-reporting-summary-flat.pdf

Life sciences study design

All studies must disclose on these points even when the disclosure is negative.

Sample size	Sample size was calculated by using G Power. Available at https://www.psychologie.hhu.de/arbeitsgruppen/allgemeine-psychologie-und-arbeitspsychologie/gpower
Data exclusions	No data was excluded.
Replication	Experiments were reproduced at least once to confirm results.
Randomization	Mice were randomly assigned to experimental groups.
Blinding	The investigators were blinded where appropriate.

Reporting for specific materials, systems and methods

We require information from authors about some types of materials, experimental systems and methods used in many studies. Here, indicate whether each material, system or method listed is relevant to your study. If you are not sure if a list item applies to your research, read the appropriate section before selecting a response.

Materials & experimental systems

n/a	Involved in the study
☒	☐ Antibodies
☒	☐ Eukaryotic cell lines
☒	☐ Palaeontology and archaeology
☐	☒ Animals and other organisms
☒	☐ Clinical data
☒	☐ Dual use research of concern

Methods

n/a	Involved in the study
☒	☐ ChIP-seq
☒	☐ Flow cytometry
☒	☐ MRI-based neuroimaging

Animals and other research organisms

Policy information about studies involving animals; ARRIVE guidelines recommended for reporting animal research, and Sex and Gender in Research

Laboratory animals	Male mice aged 7-12 weeks, wild-type C57BL/6J and αMHC-Cre bred with CITED4 floxed mice to create CITED4 knock out mice.
Wild animals	The study did not involve wild animals.
Reporting on sex	Only male mice were used to avoid possible sex-specific variance of exercise. Possible sex differences will be explored in future studies.
Field-collected samples	The study did not involve samples collected from the field.
Ethics oversight	All animal experiments were approved by the Institutional Animal Care and Use Committee (IACUC) of Massachusetts General Hospital and the Massachusetts General Hospital Center for Comparative Medicine (CCM).

Note that full information on the approval of the study protocol must also be provided in the manuscript.
